# MCDA from a health economics perspective: opportunities and pitfalls of extending economic evaluation to incorporate broader outcomes

**DOI:** 10.1186/s12962-018-0118-7

**Published:** 2018-11-09

**Authors:** Mark Jit

**Affiliations:** 1 Department of Infectious Disease Epidemiology, London School of Hygiene and Tropical Medicine, Keppel Street, London, WC1E 7HT UK; 2 Modelling and Economics Unit, Public Health England, 61 Colindale Avenue, London, NW9 5EQ UK

**Keywords:** Multi-criteria decision analysis, Economic evaluation, Equity, Deliberative process

## Abstract

**Background:**

Multi-criteria decision analysis (MCDA) is a structured decision-making process that offers greater flexibility to incorporate multiple objectives than cost-effectiveness analysis or benefit–cost analysis.

**Conclusions:**

The flexibility of MCDA requires careful consideration of its methodological underpinnings, analytical forms and cognitive biases that may arise in eliciting trade-off. The methodology of MCDA should ideally incorporate both deliberative and technical processes.

## Background

Multi-criteria decision analysis (MCDA) is a structured process for making decisions that involve several objectives. A major attraction of MCDA in health is the opportunity to extend economic evaluation methods such as cost-effectiveness analysis (CEA) and benefit–cost analysis (BCA) that are used to prioritise health care interventions. CEA in particular is widely used to inform healthcare investment decisions in many countries, particularly in the form of cost-utility analysis where results are expressed in terms of the marginal cost of an intervention per unit of a health-related utility measure (such as disability-adjusted or quality-adjusted life years). However, traditional CEA has been criticised for ignoring key considerations, such as concern for the distribution of health [1, 2]. Decision makers may wish to prioritise health gains in populations with poorer health, poorer access to health care, greater socioeconomic deprivation, or greater risk of medical-related catastrophic expenditure or impoverishment. Other beneficial aspects of health technologies that are often poorly captured in traditional CEA include encouraging medical innovation, providing peace of mind and stimulating macroeconomic growth [3].

One way to incorporate many such considerations is to use a different form of economic evaluation called BCA [4]. In this analysis, all (in principle) or some (in practice) health and non-health benefits can be valued based on their net contribution to the welfare of individuals in society, measured on the basis of the preferences and choices that those individuals make. This has the additional benefit of allowing comparisons with interventions in non-health sectors. However, for some this does not go far enough in fully accounting for the worth that society collectively places on values such as equity, liberty and freedom from exploitation [5]. Furthermore, both CEA and BCA face challenges over their legitimacy when used for priority setting, since the negative consequences of failing to spend on healthcare are usually clear and personal, while the opportunity costs of spending may be less obvious [6].

MCDA offers a framework that can capture a wider range of objectives, offer flexibility in the way trade-offs are made between competing objectives, and allow wider public participation in determining these trade-offs. It can be regarded as an extension of CEA to include a basket of objectives (not just health) without making a priori assumptions about the relative weight of each of them. Indeed, MCDA in its broadest sense is already widely practiced. Few countries base health investment decisions on the outcome of a CEA alone; other factors such as feasibility, acceptability and impact on health disparities are also considered in health technology assessments. Table 1 compares key features of CEA, BCA and MCDA.

**Table 1:** Comparison of key features of cost-effectiveness analysis (CEA), benefit–cost analysis (BCA) and multi-criteria decision analysis (MCDA)

	CEA	BCA	MCDA
Benefits included	Health and direct economic consequences of changes in health (such as healthcare spending and productivity loss)	All health and non-health benefits (in practice, only a subset of them may be feasible to include)	All health and non-health benefits that are deemed important
Outcome of analysis	Ratio between net costs and net health gains	Ratio between monetised benefits and monetised costs	Multiple outcomes representing desirable objectives. They can in principle be integrated into a single outcome (e.g. by taking a weighted sum)
How the trade-off between health and consumption is expressed	Cost-effectiveness threshold, the maximum consumption that is judged to be worth foregoing to improve a unit of health	Individual willingness to pay to avoid loss of health	Explicit or implicit tariffs between different objectives
Source of values for the trade-off	Societal judgment, as expressed through a budget limit, an economic reference case, a committee’s deliberations or other means	Individual stated or revealed preferences	Values elicited from stakeholders or members of the public

However, the very flexibility of MCDA requires careful consideration of its methodological underpinnings, particularly in two aspects discussed below.

## The relationship between MCDA inputs

Different forms of economic evaluation offer alternative frameworks for valuing the trade-off between health and consumption, to select a preferred option. BCA aims to trade consumption for health in such a way as to maximise individual welfare, in line with standard welfare economics principles such as consumer sovereignty. CEA is seen as “extra-welfarist” because it draws on alternative sources of value for the trade-off between health and consumption besides individual preferences.

MCDA also involves trading off different objectives, potentially including health, consumption and other desirable outcomes such as equity. The tariffs for these trade-offs are usually elicited from a group of participants, either explicitly through a scoring process or implicitly through consensus discussions. In this sense, MCDA can be regarded as an extra-welfarist approach, but one that potentially admits a larger universe of objectives than CEA.

Regardless of the approach taken, MCDA requires a conceptual framework that is cognisant of the relationship between its inputs. In particular, the individual elements of MCDA should be genuinely orthogonal (non-overlapping). For instance, disease burden and the output of a CEA are non-orthogonal inputs, since CEA already uses disease burden as one of its inputs. The danger of violating orthogonality is not merely theoretical. Since disease burden is an input into CEA, including both as separate entities in MCDA would lead to burden being counted twice. Participants could be told to discount its contribution when weighing the importance of the CEA, but this would be a challenging mental activity given the complexity of the computations involved in a typical CEA.

## Translating stakeholder preferences into relative weights for MCDA objectives

Most MCDA methodologies involve eliciting trade-offs between objectives. However, scores obtained are sensitive to the way (and even order) questions are framed, the number and range of options for each question and the method used to aggregate scores across participants. An alternative approach is to reach consensus through a deliberative process, but this in turn is influenced by the composition of the group and the personalities within it (such as their assertiveness, persuasiveness and perceived importance). The influence of selection, cognition and deliberation biases in elicitation exercises has been well studied [7], so it is instructive to draw on lessons from similar processes such as convening citizen juries and establishing tariffs for quality of life weights.

MCDA should ideally involve both deliberative and technical processes, combining them in such a way as to minimise the weaknesses of both processes. Stakeholder participation is arguably most important for establishing the principles that undergird any MCDA approach, such as distributional justice, liberty and autonomy. Once these principles and how they relate to each other are established, translating them into quantitative trade-offs between MCDA objectives while taking into account constraints like the size of the overall budget becomes a technical process. This translation is not value-free and hence must continue to be scrutinised and challenged by stakeholders within a process that is (perceived to be) transparent and reasonable [8]. In addition, some of these principles (such as public acceptability and avoiding discriminating) may be hard to quantify, so stakeholder input is important to ensure they are appropriately accounted for. However, a mechanical process of eliciting trade-offs or selecting investment options at this stage may miss the fundamental purpose of stakeholder participation in the MCDA exercise.

## Conclusions

MCDA offers a way to extend CEA to account for a wider variety of non-health benefits, while allowing greater flexibility than BCA to account for the way that society collectively would like to make trade-offs between competing goals such as efficiency, equity, liberty and freedom from exploitation. However, the flexibility of MCDA requires careful consideration of its methodological underpinnings, analytical forms and cognitive biases that may arise in eliciting these trade-offs.
